# Analysis of risk factors of prolonged mechanical ventilation in patients with severe burn injury

**DOI:** 10.1111/crj.13673

**Published:** 2023-07-31

**Authors:** Kui Xiao, Wen Xin Chen, Xiao Jian Li

**Affiliations:** ^1^ Department of Burn and Plastic Surgery, Guangzhou Red Cross Hospital Jinan University Guangzhou China; ^2^ Hengyang Central Hospital Hengyang China

**Keywords:** albumin, lactic acid, mechanical ventilation, severely burned patients

## Abstract

**Background:**

Mechanical ventilation is an essential means of life support for patients with severe burns. However, prolonged mechanical ventilation (PMV) increases the incidence of complications and length of hospital stay. Therefore, studying the risk factors of mechanical ventilation duration is of great significance for reducing the duration of mechanical ventilation, reducing related complications, and improving the success rate of severe burn treatment.

**Method:**

This study was a retrospective study of patients with burns ≥30% of the area admitted to the BICU of Guangzhou Red Cross Hospital affiliated with Jinan University from January 2016 to January 2023 who were mechanically ventilated. Patients were classified into the prolonged mechanical ventilation group if they were mechanically ventilated for ≥21 days. Then, independent risk factors for prolonged mechanical ventilation were determined by logistic regression analysis of the collected data.

**Result:**

Of all the 112 enrolled patients, 79 had prolonged mechanical ventilation, with an incidence of 70.5%. Logistic regression analysis revealed that including abbreviated burn severity index (ABSI%) (*P* < 0.001), moderate and severe inhalation injury (*P* = 0.005, *P* = 0.044), albumin (*P* = 0.032), lactic acid (*P* < 0.001) were independent risk factors for prolonged mechanical ventilation. In addition, ventilator‐related complications were 44% in the PMV group and 21% in the non‐PMV group.

**Conclusion:**

ABSI%, inhalation injury, albumin, and lactic acid on admission are the risk factors for PMV in severe burn patients. In addition, ventilator‐related complications were higher in group PMV than in group non‐PMV in our study.

## Graphical abstract

ABSI%, inhalation injury, albumin, and lactic acid on admission are the risk factors for PMV in severe burn patients. Early identification of risk factors for prolonged mechanical ventilation is more meaningful for clinical workers in the off‐line time selection and the prevention of complications and for managers to solve the problem of resource allocation better.

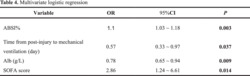

## 1 INTRODUCTION

Burns is a significant public health problem in the world. In 2011, burns killed more than 300 000 people worldwide.^1^ In a 2018 World Health Organization report, as many as 180 000 died from burns.^2^ Over the last decade, survival from a burn injury has significantly improved, partly attributed to improvements in BICU medical equipment and intensive care techniques. However, mechanical ventilation is still a life‐saving intervention for critically ill patients.^3^, ^4^ In a recent study, prolonged mechanical ventilation significantly extended the length of intensive care and hospital stay for burn patients.^5^, ^6^ The leading causes were the weakness of the respiratory muscles, ventilator‐related pneumonia, lung injury, and other related complications caused by prolonged mechanical ventilation.^7^, ^8^, ^9^ Regarding prolonged mechanical ventilation, independent risk factors have been reported in many studies, including patients with various types of surgery^10^, ^11^ and critically ill patients.^12^ However, to our knowledge, no studies have illustrated the risk factors of prolonged mechanical ventilation in severe burn patients. Therefore, in our study, we conducted a retrospective study to outline the risk factors of prolonged mechanical ventilation patients so that clinicians can be more valuable in selecting ventilator weaning time and preventing and treating complications.

## 2 SUBJECTS AND METHODS

### 2.1 Patients

**FIGURE 1:**
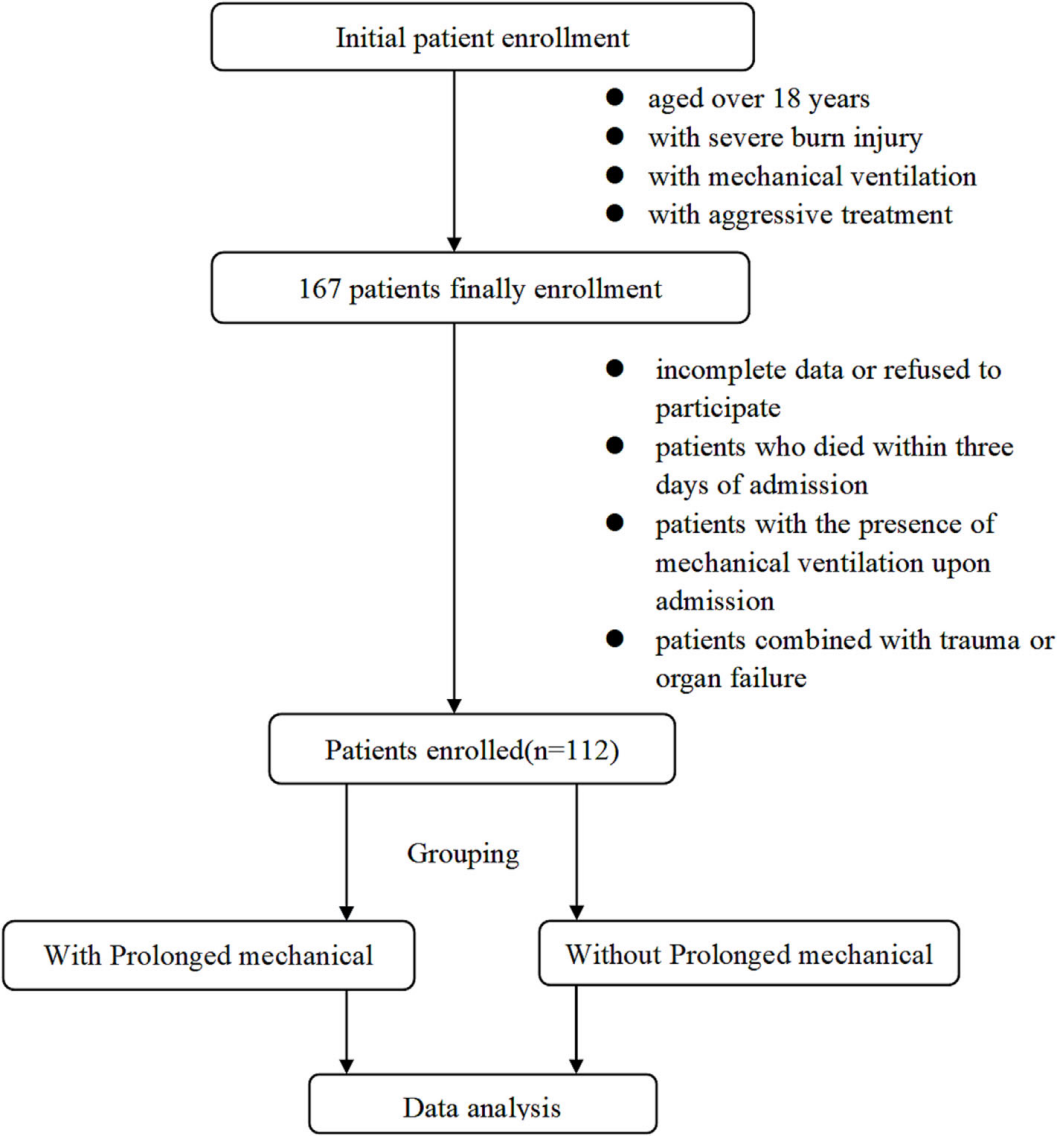
Flow diagram of patient selection.

This retrospective cohort study was based on a chart review of all burn patients admitted to the Department of Burn and Plastic Surgery in Guangzhou Red Cross Hospital affiliated with Jinan University over 7 years (January 2016 to January 2023). Adult patients aged ≥18 years with a total body surface area (TBSA%) ≥30%, requiring mechanical ventilation and aggressive treatment, were included in this study. Exclusion criteria were as follows: (1) Incomplete data or refusal to participate, (2) patients who died within 3 days of admission, (3) patients with the presence of mechanical ventilation upon admission, and (4) patients combined with trauma or organ failure (Figure 1).

### 2.2 Definition and data collection

The endpoint of the study was mechanical ventilation for more than 21 days. Mechanical ventilation time was defined as the time between the patient's first endotracheal intubation or tracheotomy after the burn and complete withdrawal from mechanical ventilation. Prolonged mechanical ventilation (invasive and noninvasive) was defined as mechanical ventilation ≥6 h/day for ≥21 days.^13^, ^14^

Patients are eligible for extubation if they achieve sufficient oxygenation and ventilation indices on blood gas analysis, maintain hemodynamic stability without needing low doses of vasoactive drugs, and exhibit typical neurological manifestations. The attending burn intensive care physician decides to extubate, re‐intubate, or perform a percutaneous tracheotomy.

ABSI%, inhalation injury, albumin, and lactic acid on admission are the risk factors for PMV in severe burn patients. In addition, ventilator‐related complications were higher in group PMV than in group non‐PMV in our study.

For patients with potential airway obstruction, preventive tracheotomy should be performed as early as possible and before the peak of tissue edema to avoid severe neck edema, which may lead to unclear anatomical layers and complex operations for those who have been intubated for 3 to 4 days and still need to use an artificial airway, tracheotomy, and intubation can be used. In an emergency, a tracheotomy should be performed immediately. In other cases, tracheotomy can be performed in time according to the patient's condition.

The following clinical data were obtained at admission, including age, sex, pre‐existing comorbidity (diabetes, hypertension, other cardiovascular diseases, and disease of the respiratory system), time of admission, body mass index (BMI), TBSA%, ABSI%, cause of injury, the severity of inhalation injury, chronic health evaluation II (APACHE II) score, and SOFA score, PaO_2_/FiO_2_ ratio. Laboratory indicators at the first admission were also collected, including lactic acid, albumin, N‐terminal pro‐B‐type natriuretic peptide (NT‐proBNP), red blood count (RBC), creatinine (Cr), blood platelet count (PTL), neutrophils, hemoglobin (Hb), C‐reactive protein (CRP), serum sodium (Na^+^), and high‐sensitivity troponin (HS‐cTn).

### 2.3 Statistical analysis

Continuous variables were compared by unpaired two‐tailed *t*‐test, expressed as mean ± standard deviation (SD), or by Wilcoxon rank sum test and median (P25–P75). Categorical variables were compared by the chi‐square test and are expressed as percentages. Univariate logistic regression analysis assessed associations between prolonged mechanical ventilation and variables. Variables that appeared to be related in the univariate logistic regression analyses with a *P* value of <0.05 were considered in multivariate regression models. Data analysis was performed using the RMS package of R.

## 3 RESULTS

### 3.1 Information about the patient

From January 2016 to January 2023, 920 patients with severe burns were admitted to the BICU of Guangzhou Red Cross Hospital affiliated with Jinan University; after final inclusion and exclusion, we analyzed data from only 112 patients, accounting for 12.1% of the total number. Seventy‐nine patients (70.5%) were under prolonged mechanical ventilation. The median (IQR) ABSI% of the prolonged mechanical ventilation group was 70.00 (55.0, 82.0); patients with moderate or severe inhalation injuries accounted for 98.7%. the mean ± D lactic acid was 6.45 ± 4.45, and the mean ± SD albumin was 26.73 ± 7.86. The specific demographic and clinical characteristics are detailed in (Tables 1 and 2).

**TABLE 1:** Clinical parameters associated with prolonged mechanical ventilation in severe burns.

Characteristics	Prolonged mechanical ventilation		*P* value
	Yes (*N* = 79)	No (*N* = 33)	
Age	44.75 ± 13.76	45.71 ± 11.42	0.314
Gender			0.65
Male	65 (85.14)	29 (88)	
Female	14 (14.86)	4 (12)	
BMI	24.43 ± 3.68	23.78 ± 2.45	0.46
Admission (h)	7.45 ± 7.56	7.61 ± 7.11	0.47
TBSA%	80 (70, 90)	50 (40, 70)	**<0.001**
ABSI%	70 (55, 82)	40 (30, 49)	**<0.001**
APACHEII score	8.45 ± 3.89	7.5 ± 2.34	0.14
SOFA score	4.56 ± 2.31	4.81 ± 1.94	0.34
PaO_2_/FiO_2_ ratio (mmHg)	267 (244, 319)	288 (247, 324)	0.377
**Inhalation injury**			**<0.001**
Mild	1 (1.3)	11 (33)	
Moderate	29 (37)	20 (61)	
Severe	49 (62)	2 (6.1)	
**Burn mechanism**			0.832
Flame	68 (86)	30 (91)	
Hot liquid	6 (7.6)	1 (3)	
Chemistry	1 (1.3)	0 (0.00)	
Electrical burn	4 (5.1)	2 (6.1)	
**Diseases in medical history**			
Diabetes	1 (1.26)	1 (3.00)	0.955
Hypertension	5 (6.30)	7 (21.00)	1.000
Other cardiovascular diseases	1 (1.26)	1 (3.00)	1.000
The disease of the respiratory system	1 (1.26)	1 (3.00)	1.000

*Note*: Data are presented as mean ± SD, or number (%). Bold values indicate *P* ≤ 0.05.

Abbreviations: ABSI, abbreviated burn severity index; BMI, body mass index; TBSA, total body surface area.

**TABLE 2:** Laboratory tests associated with prolonged mechanical ventilation in severe burns.

Characteristics	Prolonged mechanical ventilation		*P* value
	Yes (*N* = 79)	No (*N* = 33)	
**Laboratory examination**			
Lac (mmol/L)	6.45 ± 4.45	5.14 ± 2.11	**<0.001**
Alb (g/L)	26.73 ± 7.86	36.61 ± 4.11	**<0.001**
NT‐proBNP (pg/mL)	175.49 ± 238.03	43.98 ± 45.27	**0.041**
RBC (10^12^/L)	5.47 ± 1.34	5.67 ± 4.74	0.876
Cr (μmol/L)	105.40 ± 66.06	85.35 ± 26.33	0.132
Neutrophils (10^9^/L)	23.54 ± 9.37	19.17 ± 8.25	0.124
PLT (10^9^/L)	306.15 ± 169.46	296.45 ± 121.05	0.658
Hb (g/L)	175.73 ± 35.11	168.26 ± 19.67	0.383
CRP (mg/L)	25.07 ± 36.58	15.97 ± 35.45	0.415
Na^+^ (mmol/L)	137.51 ± 7.86	145.43 ± 6.16	**<0.001**
HS‐cTn (ng/L)	0.01 ± 0.11	0.07 ± 0.18	**<0.001**

*Note*: Bold values indicate *P* ≤ 0.05.

Abbreviations: Alb, albumin; Cr, Creatinine; CRP, C‐reaction protein; Hb, hemoglobin; HS‐cTn; high‐sensitivity troponin; Lac, lactic acid; Na^+^, serum sodium; NT‐proBNP, N‐terminal pro‐B‐type natriuretic peptide; PLT, platelet count; RBC, red blood cell count.

### 3.2 Variables influencing prolonged mechanical ventilation

First, the univariate logistic regression analysis showed the following: admission (OR = 1.01, 95% CI: 1.00–1.01), TBSA% (OR = 1.02, 95% CI: 1.01–1.02), ABSI% (OR = 1.02, 95% CI: 1.02–1.02), moderate inhalation injury (OR = 1.66, 95% CI: 1.32–2.10), severe inhalation injury (OR = 2.4, 95% CI: 1.91–3.03), Alb (OR = 0.91, 95% CI: 0.89–0.95), Lac (OR = 1.13, 95% CI: 1.09–1.17), Na^+^ (OR = 1.03, 95% CI: 1.02–1.04), and HS‐cTn (OR = 2.27, 95% CI: 1.42–3.63) (Table 3). Then, significant variables (*P* ≤ 0.05) from the univariate logistic regression analysis were then included in the multivariate logistic regression analysis. Our analysis shows the following: ABSI% (OR = 1.02, 95% CI: 1.01–1.03), moderate inhalation injury (OR = 1.3, 95% CI: 1.09–1.56), severe inhalation injury (OR = 1.25, 95% CI: 1.01–1.54), Alb (OR = 0.75, 95% CI: 0.65–0.94), and Lac (OR = 1.05, 95% CI: 1.02–1.08) (Table 4).

**TABLE 3:** Univariate logistic regression.

Variable	OR	95% CI	*P*
Age	1	0.99–1.00	0.314
Male	1.09	0.86–1.37	0.466
Admission (h)	1.01	1.00–1.01	**0.046**
TBSA%	1.02	1.01–1.02	**<0.001**
ABSI%	1.02	1.02–1.02	**<0.001**
Moderate inhalation injury	1.66	1.32–2.10	**<0.001**
Severe inhalation injury	2.40	1.91–3.03	**<0.001**
Alb (g/L)	0.91	0.89–0.95	**0.026**
Lac (mmol/L)	1.13	1.09–1.17	**<0.001**
Na^+^ (mmol/L)	1.03	1.02–1.04	**0.045**
High‐sensitivity troponin (ng/L)	2.27	1.42–3.63	**0.001**

*Note*: Bold values indicate *P* ≤ 0.05.

Abbreviations: CI, confidence interval; OR, odds ratio.

**TABLE 4:** Multivariate logistic regression.

Variable	OR	95% CI	*P*
ABSI%	1.02	1.01–1.03	**<0.001**
Moderate inhalation injury	1.3	1.09–1.56	**0.005**
Severe inhalation injury	1.25	1.01–1.54	**0.044**
Alb (g/L)	0.75	0.65–0.94	**0.032**
Lac (mmol/L)	1.05	1.02–1.08	**<0.001**

*Note*: Bold values indicate *P* ≤ 0.05.

Abbreviations: CI, confidence interval; OR, odds ratio.

### 3.3 Comparison of ventilator‐related complications and mortality

**FIGURE 2:**
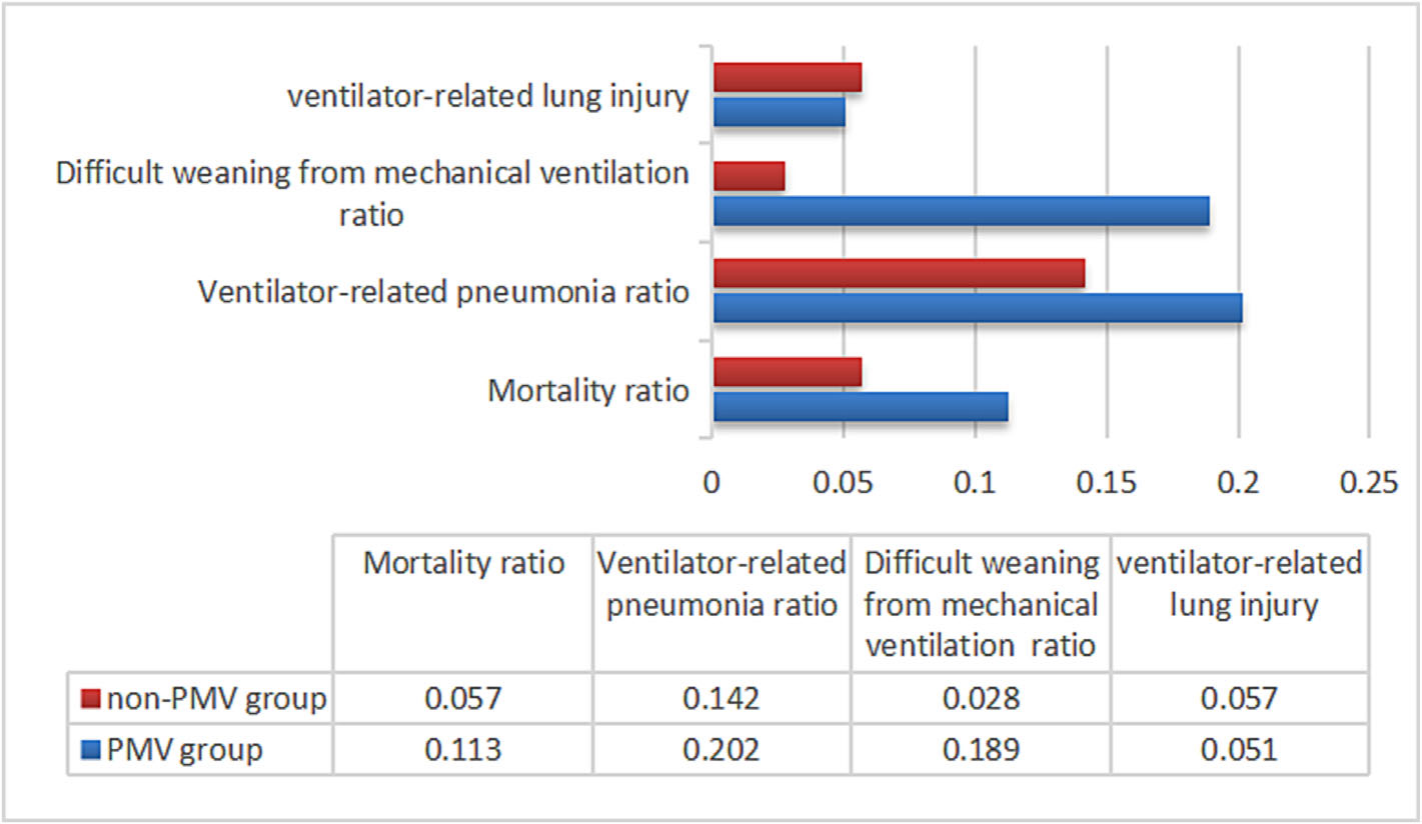
The incidence of death and ventilator‐related complications. Note: PMV group, prolonged mechanical ventilation group.

In this study, the mortality and ventilator‐related complication rates in group PMV were 11.3% and 44.2%. The mortality and ventilator‐related complication rates in group non‐PMV were 5% and 22% (Figure 2).

## 4 DISCUSSION

The present study elicited two main findings. First, we show that ABSI%, inhalation injury, albumin, and lactic acid are the risk factors of mechanical ventilation. Second, it showed that ventilator‐related complications and mortality were higher in the PMV group than in the non‐PMV group. Most retrospective studies have studied the risk factors for prolonged mechanical ventilation in critically ill or after‐surgery patients. No literature studies the risk factors for prolonged mechanical ventilation in severe burn patients. Still, it is of more positive significance to study the risk factors of prolonged mechanical ventilation in adult patients with severe burns; further early intervention, the incidence of complications of mechanical ventilation in burn patients can be reduced, and the success rate of treatment can be improved. Therefore, this article explores the risk factors of prolonged mechanical ventilation.

The PaO_2_/FiO_2_ ratio is often one of the indicators to determine whether a critically ill patient is under prolonged mechanical ventilation.^15^, ^16^, ^17^ The findings showed that early PaO_2_/FiO_2_ was not a risk factor for prolonged mechanical ventilation. These rather intriguing findings could be attributed to as follows: First, lower oxygenation in the early stage of severe burn patients is mainly caused by upper respiratory tract obstruction, but respiratory tract obstruction is less likely to occur within 12 h after inhalation injury or facial and neck ring burn injury. Most patients in our study were admitted to the hospital within 12 h after injury, and PaO_2_/FiO_2_ ratio index is not well reflected. Second, patients tend to hyperventilate in the early stage due to pain and stress response. The PO_2_ will further increase, and the oxygenation index will not be shallow. This shows the early oxygenation index of the prolonged mechanical ventilation group was not a risk factor compared with that of the nonprolonged mechanical ventilation group. In conclusion, the early oxygenation index is not a good indicator of the duration of mechanical ventilation in severe burn patients.

Second, in studies on prolonged mechanical ventilation in critically ill patients, the APACHE II score and SOFA score have diametrically opposite views on whether they are risk factors for prolonged mechanical ventilation.^18^, ^19^, ^20^ In this study, we believe that the two scores cannot predict whether the duration of mechanical ventilation will be prolonged in severe burn patients. First, the acute physiology component of the APACHE II score was an important factor in determining the duration of mechanical ventilation. While the acute physiology component does include respiratory physiology variables, it also includes non‐respiratory variables that are not part of the traditional weaning index. Second, 48 h after the patient's injury is mainly the fluid leakage period, generally after 12 h to gradually peak. After the patient's injury on the way to the hospital through oral or infusion of some crystals, adequate early blood volume circulation has no significant impact. In summary, the early APACHE II score and SOFA score cannot sufficiently reflect the mechanical ventilation time in the later period.

Third, some prospective studies have shown that the time to ventilation is associated with mild to moderate inhalation injury but not severe inhalation injury.^21^, ^22^ Furthermore, in children with burn and inhalation injuries, further prolonged ventilation time has been shown.^23^, ^24^ Regression analysis showed that moderate and severe inhalation injuries were risk factors rather than mild to moderate ones. The reasons are as follows: The judgment of inhalation injury is based on the location of bronchial mucosal injury under the branch fiber microscope. The site of severe inhalation injury is often below the bronchus, and the lung injury is relatively severe and is prone to ARDS, so the mechanical ventilation time often needs to be extended.

Fourth, obesity is an independent risk factor for prolonged mechanical ventilation. BMI was not statistically significant in this study.^25^, ^26^, ^27^ We believe that patients with severe burns are in a high metabolic state and lose weight significantly quickly, a dynamic process with no reference value.

Finally, other self‐measured gender, age, hypertension, diabetes, and respiratory diseases were not risk factors in this study. The reason is that the age of severe burn patients is mainly young and middle‐aged men around 40–60, and the incidence of primary diseases is low.

Berndtson et al. used ABSI% to predict the duration of ventilation in pediatric burn patients, and the larger the ABSI%, the longer the duration of mechanical ventilation.^28^ We studied adult patients with severe burns, but the results were consistent. Furthermore, studies have shown that the correlation of albumin and the lactic acid at admission as a correlate of complication rate, mortality, and length of stay in burn patients has been widely demonstrated.^29^, ^30^, ^31^, ^32^, ^33^ As previously mentioned, the longer the duration of mechanical ventilation in burn patients, the higher the incidence of complications, the higher the mortality rate, and the longer the length of hospital stay. Therefore, there seems to be a specific correlation between albumin and lactic acid and prolonged mechanical ventilation, consistent with the present study's findings. In the present study, prolonged mechanical ventilation was often associated with increased complication rates and mortality. Predicting whether mechanical ventilation is prolonged positively reduces or prevents ventilator‐related complications.

We conclude that ABSI%, inhalation injury, albumin, and lactic acid are risk factors for prolonged mechanical ventilation. Ventilator‐related complications were higher in group PMV than in group non‐PMV. These results suggest that the higher the burn index, the more severe the inhalation injury and the higher the possibility of prolonged mechanical ventilation. Medical workers should pay more attention to ventilator‐related complications in patients with high burn index or severe inhalation injuries. Second, ABSI% and inhalation injury cannot be intervened early. However, early clinicians should pay attention to optimizing albumin and lactate levels.

There are some limitations to the study. First, this study was a single‐center retrospective study with uneven data collection and insufficient sample size. Therefore, we recommend conducting a multicenter prospective cohort study to increase the sample size and other relevant data collection. These data were obtained from the largest burn treatment center in southern China. Second, our study lacked other variables, including those related to the treatment process (use of sedative and analgesic drugs and ventilator parameters), which were not readily available when the patients were first admitted to the hospital and were subject to uncertainty during treatment. Our study elucidates the factors associated with PMV in severe burn patients and compares the status of PMV versus non‐PMV ventilator‐related complications and mortality. Early prediction of PMV will be a top priority to improve patient outcomes and minimize healthcare costs and resource consumption.

## 5 CONCLUSIONS

In conclusion, ABSI%, inhalation injury, albumin, and lactic acid are the risk factors of prolonged mechanical ventilation. Further studies should be conducted to identify risk factors for prolonged mechanical ventilation with various respiratory pathophysiologies and extrapulmonary medical conditions to establish treatment strategies to improve the clinical outcomes and reduce the socioeconomic burden of prolonged weaning, however.

## AUTHOR CONTRIBUTIONS

*Conception and design of the study*: Kui Xiao, Wen Xin Chen, and Xiao Jian Li. *Acquisition of data*: Kui Xiao and Wen Xin Chen. *Analysis and interpretation of data*: Kui Xiao. *Drafting the manuscript*: Kui Xiao and Wen Xin Chen. *Revising the manuscript critically for important intellectual content*: Xiao Jian Li. *Approval of the version of the manuscript to be published*: Kui Xiao, Wen Xin Chen, and Xiao Jian Li.

## CONFLICT OF INTEREST STATEMENT

The authors declare no potential conflicts of interest concerning this article's research, authorship, and/or publication.

## ETHICS STATEMENT

The medical ethic committee of Guangzhou Red Cross Hospital approves this study. Each patient signed informed consent at the initiation of diagnosis, allowing for further clinical research using the clinical records.

## AUTHORSHIP STATEMENT

All persons who meet authorship criteria are listed as authors, and all authors certify that they have participated sufficiently in the work to take public responsibility for the content, including participation in the concept, design, analysis, writing, or revision of the manuscript. Furthermore, each author certifies that this material or similar material has not been and will not be submitted to or published in any other publication before its appearance in the Clinical Respiratory Journal.

## Data Availability

The data that support the findings of this study are available on request from the corresponding author. The data are not publicly available due to privacy or ethical restrictions.
